# What predicts emotional response in men awaiting prostate biopsy?

**DOI:** 10.1186/s12894-018-0340-9

**Published:** 2018-04-24

**Authors:** AnnMarie Groarke, Ruth Curtis, Deirdre M. J. Walsh, Francis J. Sullivan

**Affiliations:** 1 School of Psychology, National University of Ireland, Galway, Ireland; 2 Prostate Cancer Institute, National University of Ireland Galway, Galway, Ireland

**Keywords:** Prostate biopsy, Stress, Self-efficacy, Sense of coherence, Emotional adjustment

## Abstract

**Background:**

Incidence of prostate cancer is increasing as opportunistic screening becomes widespread and life expectancy rises. Despite screening availability, research reveals conflicting results on medical outcomes, for example, disease specific mortality. However the gold standard in early diagnosis of potentially curable organ confined prostate cancer is transrectal ultrasound-guided systematic prostate biopsy (TRUS-BX). While focus has been given to medical sequalae there is a paucity of research on the psychological impact of biopsy. Awaiting biopsy may be inherently stressful but no studies to date, have assessed men’s perception of stress and its impact on emotional response. This study, therefore, examines the role of stress and also personal resources namely, self-efficacy and sense of coherence in emotional adjustment in men awaiting a prostate biopsy.

**Methods:**

Men attending a Rapid Access Prostate Cancer Clinic for a transrectal prostate biopsy (*N* = 114) participated in the study. They completed self report questionnaires on perceived stress (PSS), generalised self-efficacy (GSES), and sense of coherence (SOC). Adjustment was measured by the Profile of Mood States (POMS-B) which assesses tension, depression, anger, fatigue, confusion and vigour.

**Results:**

Hierarchical regression analyses demonstrated that the set of predictors accounted for 17%–34% of variance across six mood states and predicted 46% of total mood disturbance. Perceived stress explained variance on all domains (11%–26%) with high stress linked to poor functioning.

**Conclusion:**

Perceived stress was the strongest and most consistent predictor of emotional adjustment. This is an important finding as stress appraisal has not been examined previously in this context and suggests that stress management is an important target to enhance emotional wellbeing of men attending for a prostate biopsy.

## Background

Prostate cancer is the most frequent malignant disease in older men and is the second most common cause of cancer-related death in this age group, after lung cancer [1, 2]. Prostate cancer rates in Ireland are the highest in Europe and Irish men have a 1 in 8 chance of developing prostate cancer [3].

Incidence of prostate cancer (PCa) worldwide is increasing as opportunistic screening becomes more widespread and average life expectancy rises [4]. Despite screening availability, research yields conflicting results on outcomes. A European trial examining disease specific mortality found benefits to population-based PCa screening [5, 6] but this was not replicated in a similar trial in the United States [7]. Furthermore, both over-diagnosis and resulting over-treatment are problematic sequelae of PCa screening due to low diagnostic specificity of prostate specific antigen (PSA levels) and prostatic biopsies [8, 9].

So the optimal management of screen-detected, localised prostate cancer remains controversial. However, the gold standard in early diagnosis of potentially curable and organ-confined prostate cancer is transrectal ultrasound-guided systematic prostate biopsy (TRUS-Bx), the most common form of investigation of raised PSA levels [10]. Biopsy can cause bleeding, pain, urinary and sexual symptoms [7, 11, 12] and it has been reported that up to 7% of men require hospital admission within 30 days of biopsy, most for febrile infections [13].

While considerable focus has been given to medical sequelae, there is a relative paucity of empirical data examining the psychological impact of a prostate biopsy.

Existing qualitative research assessing the subjective experience of the procedure indicates that while some men find it merely uncomfortable, others report that it is stressful, exhausting, extremely painful [14] and view it as the worst aspect of their disease experience [15]. Further reports reveal that some men express fears post biopsy, for example, that cancer cells might pass from a man to his wife during ejaculation or that biopsy might spread cancer cells to other parts of the body [10]. The extant qualitative literature, therefore, indicates that men perceive biopsy as a stressful event.

Moreover, quantitative studies demonstrate that the negative impact of prostate biopsy on patient well-being can even begin while waiting for the scheduled procedure [16, 17] and that there is increased anxiety and worry reported immediately before undergoing biopsy [18, 19] with a minority (20%) suffering high levels of distress at this time [20]. In the main, studies used the Hospital Anxiety and Depression Scale (e.g. [17, 21]), which is designed to detect clinical cases of anxiety and depression. It has been recommended that more detailed, specific measures (e.g. Profile of Mood States) would better reflect changes in mood and distress of men undergoing biopsy [20]. The current study, therefore, uses the Profile of Mood States (POMS).

Awaiting biopsy may be inherently stressful given that it is an unexpected, uncontrollable event with potential for loss and harm, an important hallmark of a stressor [22, 23]. For example, uncertainty about diagnosis was associated with greater stress in women awaiting a breast biopsy than women awaiting more invasive and risky treatments [24]. The transactional model of stress and coping [23] provides a framework for understanding the impact of this stressor on adjustment. Stressful experiences in the model are construed as person-environment transactions in which the impact of an external stressor is mediated by a person’s appraisal of the stressor and the psychological and social resources at his or her disposal. The model defines two types of appraisal, primary where the person asks himself, “is this stressful?” , and secondary where the person asks, “can I cope with this?” .

While it has been shown that emotional stress, as measured by elevated cortisol levels was higher in men called for a biopsy, than in a healthy matched control group [25], no studies to date have assessed men’s perception of stress and its impact on mood awaiting biopsy. This warrants investigation given that perceived stress has been shown to be an important predictor of adjustment in men with a diagnosis of prostate cancer [26, 27]. Similarly personal resources, such as self-efficacy [28] and sense of coherence [29] have been shown to be important variables in coping with cancer [30–32], an aspect of secondary appraisal within the transactional model of stress.

Self-efficacy is a broad disposition rooted in the person’s personality and life experience. It refers to both the perception of controllability of self-management tasks and sense of efficacy to use skills effectively under difficult circumstances [33]. Given a stressful situation, low self-efficacy can make people vulnerable to distress, whereas robust efficacy beliefs can serve to reduce anxiety [33–35]. Two studies to date have shown that self-efficacy beliefs have a positive impact on mood in men recently diagnosed with prostate cancer [26, 36] but less is known about its impact at biopsy stage. It would be of value, therefore, to examine if a sense of personal mastery enhances emotional response immediately prior to a biopsy. Sense of coherence, on the other hand, is conceptualised as a global orientation to life experience, including the degree to which life is viewed as comprehensible, manageable and meaningful. A strong sense of coherence is a health promoting factor that reflects a person’s capacity to cope with health stressors [29] and is associated with less distress in cancer [37–39]. While a low sense of coherence predicted high levels of distress in women awaiting an examination for diagnosis of breast cancer [40] it has not yet been examined in the context of prostate biopsy.

So it is of interest to ascertain if this personality variable impacts men at the earlier stage, when facing an invasive procedure with an uncertain outcome.

This study, therefore, assesses for the first time the role of perceived stress and personal resources namely, generalised self-efficacy and sense of coherence in predicting emotional adjustment, in a group of Irish men immediately prior to a prostate biopsy.

## Method

### Participants

Consecutive men attending the Rapid Access Prostate Cancer Clinic in a University –affiliated hospital over a 6 month period were eligible to participate. The study protocol was approved by the Institutional Research Ethics Committee of the University Hospital. Written informed consent was obtained from all participants. Inclusion criteria were men awaiting biopsy for the first time who had completed at least second level education. Exclusion criteria were other comorbidities. Men were asked by the researchers to complete a questionnaire in the clinic while awaiting the procedure. Out of 215 men attending, 114 agreed to participate (53% response rate). The majority who declined, said they were unable to concentrate on the task or said they wouldn’t have time before being called for the procedure.

### Materials

The Perceived Stress Scale (PSS) [41] is a 14-item scale which measures the degree to which situations in one’s life over a 1 month time frame are appraised as stressful. It taps the degree to which respondents find their life unpredictable, uncontrollable and overloaded. Respondents indicate how often they thought or felt a certain way on a five point Likert scale from 0 “never” to 4 “very often”. Scores can range from 0 to 56 with higher scores indicating more perceived stress. In this study Cronbach’s alpha coefficient was .67.

Generalised Self-Efficacy Scale (GSES) [35] includes 10 items (e.g. *‘I can always manage to solve difficult problems if I try hard enough*’) and the responses for each item range from strongly disagree (1) to strongly agree (4). The scale provides one overall summmative score and has been shown to have high reliability (α = .96). In this study Cronbach’s alpha coefficient was .71.

The Sense of Coherence (SOC-13) scale [29] has three components: Comprehensibility (items 2, 6, 8, 9, 11), Manageability (items 3, 5, 10, 13) and Meaningfulness (items 1, 4, 7, 12). This scale is rated on a 7-point likert scale, a total score can also be used and the coding for items 1, 2, 3, 7 and 10 should be reversed. SOC scores range from 13 to 91. Antonovsky [42] concluded that content, construct and criterion validity were adequate. In the current study the total scale score reliability etimate was .52.

The Profile of Mood Scale-Brief (POMS-B) [43] is a 30 item indicator of psychological state, used to measure mood disturbance across 6 dimensions: tension/anxiety, depression/dejection, anger/hostility, fatigue/inertia, confusion/bewilderment and vigour/activity. It provides individual subscale scores and a total mood disturbance score. The POMS-B is made up of the 5 highest loading items per subscale based on six previous investigations of the POMS [44]. In this study Cronbach’s alpha ranged from .60–.92. The total score reliability estimate was .92.

### Statistical analyses

The statistical program G*Power was used to conduct power analysis. Adhering to Cohen’s [45] guidelines for small (*r* = 0.02), medium (*r* = 0.15), and large (*r* = 0.35) effects, two-tailed alpha of .05 was assumed for all tests. With 4 predictors as well as a medium effect size and a power of 0.8, the recommended total sample size for linear multiple regression was 85.

Inspection of histograms and analysis of skewness showed that POMS subscales with the exception of tension and vigour were skewed. Outliers were windzorised which reduced the skewness to an acceptable level. Pearson correlation coefficients assessed relationships between predictors and outcomes. Missing data was less than or equal to 5% on all scale items (with the exception of two items where the proportion was 9.5% and 9.6%). Using Little’s MCAR test, data was found to be missing competely at random (*p* > .05) and therfore, the Expectation Maximization algorithm was used to substitute missing values [46]. EM is considered an excellent procedure for handling missing data [47, 48], and is advised when data are MCAR or MAR [49] and the percentage of missing data is minimal (i.e., less than 5%) [48, 49]. Hierarchical regression was chosen as the study is based on the transactional model of stress [23]. Accordingly, age was entered in Step 1, the effect of primary appraisal, that is level of perceived stress, was entered in Step2. The effect of secondary appraisal was examined in subsequent steps with self-efficacy entered in Step 3 and sense of coherence entered in Step 4. Self-efficacy was entered before sense of coherence as previous literature has shown a link with distress at diagnosis of prostate cancer [26, 36].

## Results

Descriptive data are presented in Table 1. The sample scored in the moderate range on perceived stress and sense of coherence and in the upper range in self-efficacy. The intercorrelations between predictors and the 6 outcomes are shown in Table 2 and reveal that relationships between stress and all mood variables are in the expected direction with stress linked to high tension, depression, anger, fatigue, confusion, and low vigour. Equally, low self-efficacy and low sense of coherence correlated with high tension, depression, anger, fatigue, confusion, and low vigour.

**Table 1:** Descriptive Statistics for predictor and outcome variable for 114 men attending for Biopsy

	Test Range	Sample Range	*M*	*SD*
Age		41–77	62.56	7.25
PSS	0–56	4–37	20.40	7.11
GSES	10–40	19–40	31.06	4.91
SOC	13–91	36–91	68.31	12.50
POMS				
Tension	0–20	0–14	5.31	4.07
Depression	0–20	0–9	2.77	3.04
Anger	0–20	0–10	2.72	3.22
Fatigue	0–20	0–15	4.53	4.14
Confusion	0–20	0–10	4.70	2.80
Vigor	0–20	0–20	9.33	4.52
POMS TMD		-15-42	10.63	15.48

*GSES* General Self-Efficacy Score. *PSS* Perceived Stress Scale. *SOC* Sense of Coherence Scale

**Table 2:** Intercorrelations between Age, Stress,Self-efficacy, Sense of coherence and Mood in 114 men attending for a bipsy

	1	2	3	4	5	6	7	8	9	10	11
1. Age	–										
2. PSS	.00	–									
3. GSES	−.03	−.49**	–								
4. SOC	.10	−.53*	.58**	–							
5. Anxiety	.00	.52**	−.36**	−.39**	–						
6. Depression	.01	.60**	−.41**	−.58**	.64**	–					
7. Anger	−.04	.513**	−.31**	−.50**	.56**	.78**	–				
8. Fatigue	.08	.48**	−.41**	−.47**	.64**	.64**	.59**	–			
9. Confusion	.01	.37**	−.30**	−.43**	.62**	.68**	.59**	.69**	–		
10. Vigor	−.20*	−.39**	.37**	.19*	−.16	−.13	−.08	−.23*	.13	–	
11. POMS TMD	.06	.64**	−.48**	−.56**	.82**	.83**	.78**	.84**	.73**	.40**	–

*Note;* * *p* < .05, ** *p* < .01, *** *p* < .001. *N* = 115. *GSES* General Self-Efficacy Score. *PSS* Perceived Stress Scale. *SOC* Sense of Coherence Scale

The results of the regression analysis are presented in Table 3. Age explained 3% of variance on vigour with older participants reporting lower vigour-activity. Age did not contribute to other mood states. Perceived stress explained variance in all domains; tension (26%), depression (26%), anger (24%), fatigue (22%) confusion (11%) and vigour (15%), with the beta weights showing relationships in expected directions. Self-efficacy explained additional variance on the domain of vigour (4%) with high self-efficacy indicative of good status on this index. Sense of coherence significantly predicted four out of six mood outcomes; depression (8%), anger (6%), fatigue (3%) and confusion (6%). In all cases beta weights showed that sense of coherence was related to poorer function.

**Table 3:** Hierarchical Multiple Regression of the Role of Age, Stress, Self-Efficacy and Sense of Coherence on Six Subscales and Total Score of the Profile of Mood States Questionnaire in 114 Men Attending for a Biopsy

Predictors	Tension – Anxiety			Depression – Dejection			Anger - Hostility			Fatigue - Inertion		
	β	Fchange	Adj R^2^ ch	β	Fchange	Adj R^2^ ch	β	Fchange	Adj R^2^ ch	β	Fchange	Adj R^2^ ch
(1) *Demographic Variables* (Age)	0.01	0.00	−0.01	0.05	0.01	−0.01	−0.01	0.18	−0.01	0.10	0.77	−0.00
(2) *Stress* (PSS)	0.42^***^	42.37^***^	0.26	0.40^***^	62.50^***^	0.26	0.36^***^	40.23^***^	0.24	0.28^**^	33.29^***^	0.22
(3) *Self-Efficacy* (GSES)	−0.08	2.02	0.01	0.01	3.20	0.01	0.06	0.75	0.00	−0.11	5.75^*^	0.03
(4) *Sense of Coherence* (SOC)	−0.12	1.21	0.00	−0.38^***^	16.22^***^	0.08	−0.34^***^	10.83^***^	0.06	−0.26^*^	6.24^*^	0.03
Total *Adj R*^2^			0.26			0.34			0.29			0.28
Predictors	Confusion - Bewilderment			Vigour – Activity			Total Mood Disturbance					
(1) *Demographic Variables* (Age)	0.04	0.01	−0.01	−0.18^*^	4.68^*^	0.03	0.08	0.43	−0.01			
(2) *Stress* (PSS)	0.19	17.74^***^	0.11	−0.31^**^	20.81^***^	0.15	0.45^***^	79.97^***^	0.40			
(3) *Self-Efficacy* (GSES)	−0.01	2.35	0.01	0.30^**^	6.22*	0.04	−0.10	7.19^**^	0.03			
(4) *Sense of Coherence* (SOC)	−0.33^**^	8.54^**^	0.06	−0.13	1.47	0.00	−0.27^**^	8.91^**^	0.04			
Total *Adj R*^2^			0.17			0.22			0.46			

*Note;* * *p* < .05, ** *p* < .01, *** *p* < .001. *N* = 115. GSES; General Self-Efficacy Score. PSS; Perceived Stress Scale. SOC; Sense of Coherence Scale. Standardised beta values are reported from the final model (Model 4). *F* change illustrates significance of each step over and above previous step

The total amount of variance explained by all predictors in the model for each mood index was as follows; Tension 26%, Depression 34%, Anger 29%, Fatigue 28%, Confusion 17%, and Vigour 22%. The model explained more than a quarter of the variance on four of the emotional states and 46% of variance on total mood disturbance.

## Discussion

This current study examined the role of predictors of emotional adjustment in men attending for a prostate biopsy. An important finding is that perceived stress was the strongest and most consistent predictor of adjustment in men awaiting a biopsy. It explained variance ranging from 11 to 26% on all six emotional domains with higher levels of stress at biopsy linked to poor adjustment. Overall it explained 40% of variance on total mood distubance. This is in line with the finding by Curtis et al. [26] and Orom et al. [36] in men with newly diagnosed prostate cancer and so illustrates for the first time a similarly strong impact of stress in predicting mood states during the biopsy phase.

Of particular interest is that while stress best explained variability on tension and depression it also predicted a quarter of the variability in anger and fatigue, and over 10% of variance in vigour. This emphasises the need to extend assessment beyond anxiety and depression, which is the typical focus of adjustment in the biopsy literature, to include a more comprehensive analysis of mood in men awaiting biopsy. The PSS measured the extent to which patients found their lives uncontrollable and unpredictable within the previous month and so this suggests that the stress of waiting for a scheduled biopsy can impact on emotional responses. This concurs with previous findings [16, 17, 24] and pinpoints, moreover, the importance of adequate preparation for the upcoming procedure and its emotional aftermath [50]. Recent systematic reviews report that psychosocial interventions with men with prostate cancer have enhanced participants’ cancer knowledge, quality of life and mood [51, 52]. In light of findings here, the effect of psychosocial interventions at the biopsy stage should be investigated. Further research, differentiating the particular sources of stress, whether related to fear of the procedure and or worry about a potential diagnosis, could usefully inform the design of such interventions.

In the present study sense of coherence explained some additional variance on four of the six emotional indices (depression, anger, fatigue and confusion) with low sense of coherence linked to poor functioning similar to women awaiting biopsy [40]. Such that those who viewed life as comprehensible, manageable and meaningful experienced better mood states in the face of this challenging medical procedure. While the amount of variance explained is small, these results for men awaiting biopsy are congruent with Antonovsky’s model [29] and a recent meta-analysis [39], which purports consistency of the sense of coherence–distress association across medical subgroups of cancer patients. While further research is needed to confirm these relationships, findings suggest that the SOC scale could be a useful screening tool to detect those patients who are vulnerable and who would benefit from counselling to improve their coping capacities [38].

Self-efficacy had minimal effect on mood when awaiting biopsy but did explain a small amount of variance in domains which pertain to states concerning energy level (vigour). This is in contrast to the positive role of self-efficacy on mood (distress) in men diagnosed with prostate cancer [26, 36]. Perhaps the period prior to biopsy does not yet present symptom-related self management tasks and so self-efficacy beliefs are not as pertinent to emotional state.

Alternatively, the role of self-efficacy in adjustment may depend on the subjective appraisal of a potential diagnosis. A qualitative analysis, found that some men construed their prostate cancer as non-threatening, associated with few symptoms and a positive prognosis [15]. According to self-regulation theory [53] perceptions of an illness, for example, the extent to which it is seen as curable or incurable, can impact perceived ability to cope. Future research, could therefore, explore the interaction between threat appraisal, illness beliefs and self-efficacy to provide a more in depth understanding of emotional adjustment in men awaiting a prostate biopsy.

A limitation of the study is that it is cross-sectional and so does not allow for causal inferences among variables. Furthermore the modest sample size and response rate may influence generalisability of the findings, as may the low alpha reliability of the sense of coherence measure.

The study does, however, provide useful insight into predictors of adjustment in consecutive men attending for a biopsy at a prostate cancer clinic. It identifies that stress had an important explanatory role in mood disturbance with those higher in stress reporting poor adjustment across a range of mood states. This is an important finding as stress appraisal has not been examined previously in this context and it may be that stress management is a more important target than enhancement of personal resources to the emotional wellbeing of men attending for a biopsy. This fits with a growing consensus that intervening to reduce distress is an essential component of cancer care [54–56].

It is important that health professionals are fully cognizant of misconceptions held as well as the emotional impact of investigative procedures, such as transrectal prostate biopsy so that they can provide optimal information and psychological support at this time.
